# Data sharing among data monitoring committees and responsibilities to patients and science

**DOI:** 10.1186/1745-6215-14-102

**Published:** 2013-04-19

**Authors:** Iain Chalmers, Douglas G Altman, Hazel McHaffie, Nancy Owens, Richard WI Cooke

**Affiliations:** 1 James Lind Initiative, Summertown Pavilion, Middle Way, Oxford, OX2 7LG, UK; 2 Centre for Statistics in Medicine, University of Oxford, Wolfson College Annexe, Linton Road, Oxford, OX2 6UD, UK; 3 Freelance writer, formerly Deputy Director of Research, Institute of Medical Ethics; 4 PO Box 5080, Braddon, ACT 2612, Australia; 5 Department of Neonatal Medicine, Liverpool Women's Hospital, Crown Street, Liverpool, L8 7SS, UK

## Abstract

Over the past three decades it has become increasingly recognized that systematic assessment of as high a proportion as possible of relevant research evidence is needed to protect the best interests of patients and the public. For example, this principle is manifested in clinical guidelines and, increasingly, in the design and monitoring of new research. For scientific and ethical reasons, those responsible for monitoring the progress of ongoing clinical trials may need to seek unpublished and interim data to protect the interests of actual or potential participants in research. The challenge facing data monitoring committees has received relatively little attention, however. In this paper we review some of the commentaries on the issue and the few accounts of actual data monitoring committee experiences. We then present details of our own recent experience as members of the data monitoring committee for the BOOST-II UK trial (ISRCTN:0084226), one of five concurrent trials assessing the level of arterial oxygen which should be targeted in the care of very premature neonates. We conclude that efficient protection both of the interests of actual or potential participants in research and of science requires that data monitoring committees have access to all relevant research, including unpublished and interim data.

## Introduction

### Taking account of all relevant evidence when planning randomized trials

Science is cumulative, and scientists should accumulate scientifically. Failure to observe this principle in clinical research has resulted in substantial avoidable suffering and death, and unnecessary waste of research resources [1]. Applying this principle in practice means that clinical trials should be designed in the light of systematic reviews of relevant existing data [2].

The need for statistically more reliable clinical research findings became widely recognized in the late 1970s and 1980s, mainly among researchers assessing treatments for heart disease and cancer. This was manifested in the emergence both of systematic reviews using meta-analysis and of very large trials. In addition, trialists formed international groups to conduct collaborative analyses based on individual patient data derived from their separate but similar trials. In the 1990s, some trialists took this principle a step further by pioneering ‘prospective meta-analyses’: when similar but administratively separate trials were being conducted more or less concurrently, they agreed in advance which common core data should be collected by every team, and which collaborative analyses would be done on completion of data collection, using pooled data [3]– [5].

### Taking account of all relevant evidence when monitoring clinical trials

It is increasingly widely accepted that all relevant evidence (published and unpublished) should be taken into account when estimating treatment effects from completed trials. Likewise, there is a consensus that data monitoring committees (DMCs) for ongoing clinical trials should take account of evidence from any relevant new publications or conference presentations [6]. Indeed, if harmful effects are suspected, relevant evidence may include research on the same intervention but given for different health problems.

This principle has been codified in the DMC charter proposed by the DAMOCLES Group [6,7]. That group considered the issue of sharing information among DMCs of similar concurrent trials. They noted that the issues are complex but observed that ‘there are merits in the committee chairs making contact and agreeing to consult each other if their committee is faced by, or has decided to make, a decision that would change that trial’s protocol (including an early end to recruitment)’ [6].

In the case of the NeOProM Collaboration, discussed below, the DMC charter for the UK trial specified that the DMC's judgments should be based not only on the accumulating data from the UK trial, but also on any other relevant data. Accordingly, when one of the trials (the US trial) in the NeOProM Collaboration reported a difference in mortality of marginal statistical significance [8], the other four DMCs responsible for the related ongoing trials commissioned new analyses based on interim data from their respective studies. None of these analyses were judged to provide any grounds for discontinuing recruitment. Nevertheless, this ‘reassurance’ was insufficient to prevent some centers deciding to cease recruitment. A similar series of analyses occurred in relation to two controlled trials of ganciclovir for the prevention of cytomegalovirus disease in HIV/AIDS patients [9].

### Taking account of all relevant evidence - unpublished as well as published - when monitoring clinical trials

For scientific and ethical reasons, those monitoring the progress of any ongoing trial should base their judgments on analyses of as high a proportion as possible of the relevant data. This implies going beyond relevant evidence already in the public domain to unpublished and interim data.

More than a decade ago it was recognized that sharing data confidentially among DMCs could improve the chances that trials would be completed successfully [10]. This was indeed the case in respect of the decisions taken by a DMC responsible for two related controlled trials of radiotherapy. Taking account of interim analyses from both studies led to a recommendation that recruitment should continue and, importantly, this led to a different conclusion from the research than would otherwise have happened [11].

Sharing unpublished interim data among DMCs responsible for separate but similar trials can also lead to decisions to discontinue or to continue recruitment. For example, after DMCs monitoring three similar trials comparing different policies for managing cerebral infarcts had shared all of the accumulated data from the three trials, it was decided that recruitment to all three trials should cease [12]. By contrast, prompted by a worrying trend in one of two trials of combination antiretroviral therapy in HIV/AIDS, the two DMCs agreed to share key outcome data on the basis of strict confidentiality, and the resulting analysis provided the reassurance needed to allow continued recruitment [13].

Although it is not uncommon that there are other similar concurrent trials, most DMCs regard the particular trial that they have been appointed to monitor as ‘an island unto itself’, and they are usually disinclined to share interim results. The issue of divulging such interim data to other DMCs faced with similar ethical and scientific challenges rarely arises. There are, however, rare examples of DMCs conceptualizing their responsibilities to patients and science as requiring them to taking account of all relevant evidence - unpublished as well as published. Should DMCs take that view, however, they may well encounter major procedural and other impediments.

### A relevant case study: five concurrent trials of oxygen supplementation in preterm infants

#### Background

Our experience involved a planned prospective meta-analysis of studies recruiting concurrently to address a longstanding uncertainty about what level of arterial oxygen saturation should be targeted when prematurely-born infants require supplementary oxygen [14]. Evidence from controlled trials had shown that if the oxygen levels are relatively high there is an increased risk of the infant developing the blinding condition retinopathy of prematurity. On the other hand, observational data had suggested that keeping levels of arterial oxygen relatively low might result in increased mortality, and neurological handicap among long term survivors.

During the past decade, five similar trials - in the USA, New Zealand, Canada, Australia and the UK - were organized more or less concurrently to investigate this therapeutic dilemma. Although they were separately organized and funded, all of them compared different oxygen tension targeting strategies intended to minimize both mortality and serious morbidity. It was recognized at the outset that none of the five trials, individually, would have the statistical power to provide a reliable estimate of survival without serious morbidity 18 to 24 months after birth. Indeed, two of these trials were only funded on the understanding that the data from several similar trials would be combined. Consequently the researchers responsible for the trials formed the Neonatal Oxygenation Prospective Meta-analysis (NeOProM) Collaboration, which agreed that, on completion, data from all the trials would contribute to a collaborative analysis [15].

### Partial success in applying a good principle in practice

As members of the DMC for the UK trial contributing to the NeOProM Collaboration, we believe it is important to describe our experience of endeavoring to apply this good principle in practice. Our DMC charter stated that we had the right to share confidential information if we deemed this to be necessary. Furthermore, the NeOProM Collaboration’s protocol recognized the importance of communication among the DMCs for the related oxygen trials, and stated that its management committee would consider any requests from DMCs to share data, should the need arise [15]. Accordingly, because data from the other four trials were of obvious relevance to our responsibility, the chair of our DMC wrote to the DMC chairs of the other trials in 2006, expressing the ‘hope that we can help each other fulfil our respective commitments to the babies being treated in these trials’ (Emails sent 11 and 15 July 2006).

No response was received from the other DMC chairs for several years; but consideration of the proposal became urgent when, more than three years later, in 2009, the management group of the US trial sent results, in advance of publication [8], to those associated with the trials that were still recruiting. Although the US trial results did not have sufficient follow-up to report on the primary outcome (long term survival without major disability), they showed a marginally statistically significant (*P* < 0.04) higher level of mortality when lower levels of oxygen were targeted.

This information prompted the Canadian DMC chair to contact the UK DMC chair to ask for UK reactions to these results (Email received 10 December 2009). After each of the DMCs responsible for monitoring the trials that were still recruiting had independently considered the US results and the data from their own country, none found any grounds for possible discontinuation of recruitment.

These separate analyses of the individual oxygen trials were insufficient to quell concern among many clinicians, however. These prompted the principal investigator of the UK trial to write to the UK DMC chair (letter received 17 June 2010) to say that British clinicians wanted to know whether ‘the *aggregated* evidence to date is such that a final analysis of the full UK trial sample or *the pooled data from all of the trials* is strongly expected to show a significant difference in mortality’ (emphasis added).

The UK DMC chair responded (Email sent 23 June 2010) by reiterating his view that it was ‘in the interests of babies and their parents that we (the DMCs) should base our judgments on ALL the currently available data’. His response was copied to the Canadian and Australasian DMC chairs. The Canadian DMC chair responded positively, saying that his committee would be comfortable with a pooled meta-analysis of key outcomes (Email received 23 June 2010), and suggested that this analysis should be conducted by the statisticians of the relevant trials. He also made clear that the results of this analysis should be provided confidentially, and only to the DMCs of the respective trials for their consideration. As a result, proposed standard tabulations taking account of the analyses envisaged in the NeOProM Collaboration’s protocol were drafted and circulated (on 5 August 2010).

The encouraging signs of willingness among the DMCs responsible for ongoing trials to collaborate in pooled analyses were short lived, however. Following a meeting with the principal investigator of the Canadian trial, the chair of the Canadian DMC first informed the other two DMC chairs that the Canadian view was now that the collaborative analysis should not be conducted by the three trial statisticians working together, as he had suggested previously, but instead only by the Canadian DMC statistician (Email received 4 August 2010). The Canadian trial steering committee (TSC) issued a memorandum conveying that decision, but then sent another that concluded with its view that:

‘At this time, the risk of premature disclosure of mortality data from ongoing NeOProM trials considerably outweighs any potential benefits of any interim pooled analysis. Therefore, we will no longer authorize the release of interim COT [Canadian Oxygen Trial] data for the purpose of such a pooled analysis’ (memorandum dated 23 November 2010 received 24 November 2010).

Given that the Canadian trialists had meanwhile ceased recruitment (because they had enrolled their target sample size), the conduct of their trial could not be influenced by shared access to all the relevant data available. However, their decision not to share their data had major consequences for the two DMCs (UK and Australasian) that remained responsible for monitoring their ongoing trials. These DMCs would have been able to make more informed judgments about stopping or extending recruitment had they had access to data from the Canadian trial in addition to data from the ongoing trials for which they were responsible in their own countries.

The significance of the Canadian action was highlighted still further by the fact that, part way through the trial, there had been changes in the equipment used to monitor the interventions in the oxygen trials [16]. Based on interim analyses, there was a distinct possibility that extended recruitment (and so extra funding) would be needed to obtain sufficient evidence relevant to the modified equipment, which had been distributed worldwide to replace that used at the start of the trials.

Decisions to stop or extend recruitment in the two ongoing trials would have been better informed had the DMCs had access to the relevant data from all the relevant trials. As it was, the two DMCs were able to benefit from access to each other’s interim data and a combined collaborative analysis of these, together with the published results of the US trial [8]. Using conservative statistical assumptions, the combined analysis suggested strongly that targeting arterial oxygen to remain within the higher range was associated with a convincing, and statistically highly significant, increased survival. Each of the two DMCs decided independently to forward these pooled analyses promptly to their respective TSCs. The TSCs decided independently of each other to cease recruitment to their respective trials; and they subsequently reported this decision and the collaborative analyses which had led to their decisions in a letter published in the *New England Journal of Medicine*[17].

## Discussion

A decade ago, Dixon and Lagakos [18] noted that it is expected that DMCs will base their recommendations on ‘all available relevant information, even if they must go to some extra trouble to get it’. Given the evidence that DMC decisions based on analyses of all relevant evidence, unpublished as well as published, can be very important for patients and science, any arguments against sharing interim data confidentially among DMCs that are monitoring related trials need to be very persuasive, on both ethical and scientific grounds. Dixon and Lagakos noted that there are fewer concerns about making available to the DMCs of other ongoing trials the results of trials that have completed recruitment but remain unpublished [18].

Despite arguments against confidential data sharing among DMCs [18], our experience leads us to believe that, in the circumstances in which we found ourselves, the ethical and scientific arguments in favor of sharing data outweighed those against sharing. It is in the interests of patients, participants in clinical research and science that judgments about the initiation, analysis and reporting of clinical research should be informed by as high a proportion as possible of the relevant data. This principle has become increasingly accepted over recent years. What has been less widely considered is that judgments during a trial should also ideally be informed by analyses of all relevant interim research data [19,20]. This may be achieved by DMCs sharing confidential interim data, although this practice appears very rare. Occasionally it may result from planned publication of interim data [21].

The example we have described of partial success in applying this principle in practice raises questions about how chief investigators should view their responsibilities to patients, clinicians and science. Although frontline clinicians involved in the neonatal oxygen trials had requested collaborative analyses of all the relevant interim data, and despite agreement in principle among all the DMCs that these analyses would be undertaken and shared in confidence, this proposal was vetoed by the steering committee of a trial that had completed recruitment. We believe that this decision by the Canadian TSC was not in the interests of patients or science, quite apart from making the onerous responsibility of the DMCs for ongoing trials more burdensome than it needed to have been. This judgment reflects the fact that pre-trial selection of target sample sizes inevitably involves guesswork that interim analyses may show to have been incorrect, to which the appropriate scientific response is sample size reestimation [22].

A DMC’s responsibility for the integrity of a trial is a serious one. Decisions to provide trial steering committees with unblinded analyses of accumulated data, let alone make recommendations about whether a trial should continue, are not to be taken lightly. The availability of interim data from concurrent trials would make the task of DMCs easier. Few authors seem to have considered this issue, even those who have focused on ‘adverse events’ [23,24]. In that context, Shah *et al*. wrote: ‘DSMBs [Data Safety and Monitoring Boards] and sponsors have an ethical obligation to disclose information to protect the safety of participants in other trials [24]’.

A major overarching ethical responsibility of DMCs is to do what is best for current and future patients. We believe that judgments should be informed by as high a proportion as possible of the relevant evidence - published and unpublished. Although this principle has been stated previously, it remains far from accepted, so further discussion and debate is needed to identify any circumstances in which data sharing among DMCs would operate against the interests of patients first and science second. Consideration should be given to (i) who should have the authority to approve and to veto confidential sharing of interim data among DMCs; and (ii) how much weight to give to data from a DMC’s particular trial and data from other similar trials (especially if they have begun at different times), and whether to combine data formally using meta-analysis.

Professor Martin Dennis (Email to IC, 25 February 2013) has kindly provided us with an example of the arrangements that have been agreed among the chief investigators of three concurrent trials (in the UK, Australasia and Sweden) to establish whether giving fluoxetine for six months after acute stroke improves recovery (FOCUS, ISRCTN83290762; AFFINITY, ACTRN1261000774921; EFFECTS, not yet registered). The three trials have virtually the same protocol, although methods of follow-up and secondary data items have had to take account of different national settings. The arrangements they have agreed for data sharing provide an exemplar of how ethical and scientific responsibilities can be addressed. The chief investigators of each trial are members of the TSC of the other trials. Each trial has its own DMC but the charters are very similar. Consideration was given to routine review by a common DMC of all available data from the three trials combined, but this was judged to be impractical because of the different database systems being used. The compromise decision has been that, where stipulated by the charters, the chairs of the three respective DMCs will communicate and share information which might alter the decision making of the others. The chief investigators have also written into their protocols (which will be published) that there will be a pre-specified individual patient meta-analysis using the data from all three trials.

### Recommendations

We suggest that, when trials are being planned, the possibility of sharing information should be discussed with those responsible for any other similar, planned or ongoing trials, as the chief investigators of the three trials of fluoxetine after stroke have done. Similar good practice was exemplified by the investigators of the three trials of surgery for malignant infarction of the middle cerebral artery, to which we referred earlier in this paper [12]. They reported that:

‘a collaborative protocol for a pooled analysis of individual patient data from the three trials was planned before the interruption of the first two trials. The principal aim of this pooled analysis was to obtain sufficient data to reliably estimate the effects of early decompressive surgery as soon as possible so as to avoid unnecessary (and unethical) continuation of randomisation in the individual trials [12]’.

It is clearly scientifically and ethically desirable not only to discuss the possibility of data sharing, but also to lay down, as far as possible, how and when this should happen. Shah and colleagues proposed that ‘sponsors and DSMBs routinely work together in advance to develop a plan for disclosing relevant information’ [24]. We agree. If the academic community wishes to be seen to be putting the interests of patients and science above individual agendas, concerns and benefits, the issue of sharing interim analyses needs to be given more attention than it has received in the past. One of the challenges in taking this matter further is that perspectives are required from both ethics and science.

## Abbreviations

DMC: Data Monitoring Committee; DSMB: Data and Safety Monitoring Board; NeOProM: Neonatal Oxygenation Prospective Meta-analysis

## Competing interests

The authors declare that they have no competing interests.

## Authors’ contributions

All the authors were members of the data monitoring committee for the BOOST-II UK Trial (ISRCTN: 00842661). IC and DGA wrote the initial draft. HM, NO, and RWC provided critical comments. All authors read and approved the final manuscript

## References

[B1] Chalmers I Rothwell P The lethal consequences of failing to make use of all relevant evidence about the effects of medical treatments: the need for systematic reviews Treating individuals: from randomised trials to personalised medicine 2007 London: Lancet 37 58

[B2] Clark S Horton R Putting research into context - revisited Lancet 2010 376 10 11 10.1016/S0140-6736(10)61001-X 20609979

[B3] Simes RJ Prospective meta-analysis of cholesterol-lowering studies: the Prospective Pravastatin Pooling (PPP) Project and the Cholesterol Treatment Trialists (CTT) Collaboration Am J Cardiol 1995 76 122c 126c 10.1016/S0002-9149(99)80482-2 7572681

[B4] International Multicentre Pooled Analysis of Colon Cancer Trials (IMPACT) Efficacy of adjuvant fluorouracil and folinic acid in colon cancer Lancet 1995 345 939 944 7715291

[B5] Laupacis A Research by collaboration Lancet 1995 345 938 10.1016/S0140-6736(95)90695-9 7715290

[B6] Grant AM Sydes M McLeer SK Clemens F Altman DG Babiker A Campbell MK Darbyshire J Elbourne D Parmar M Pocock S Spiegelhalter S Walker A Wallace S 2. Issues in data monitoring and interim analysis of trials (The DAMOCLES project) Health Technol Assess 2005 9 7 1 223 15763038 10.3310/hta9070

[B7] DAMOCLES Study Group A proposed charter for clinical trial data monitoring committees: helping them to do their job well Lancet 2005 365 711 722 15721478 10.1016/S0140-6736(05)17965-3

[B8] SUPPORT Study Group Target ranges of oxygen saturation in extremely preterm infants N Engl J Med 2010 362 1959 1969 20472937 10.1056/NEJMoa0911781 PMC2891970

[B9] Hillman DW Louis TA DSMB case study: decision making when a similar clinical trial is stopped early Control Clin Trials 2003 24 85 91 10.1016/S0197-2456(02)00274-X 12559646

[B10] Ellenberg SS Independent data monitoring committees: rationale, operations and controversies Stat Med 2001 20 2573 2583 10.1002/sim.730 11523070

[B11] Parmar MKB Griffiths GO Spiegelhalter DJ Souhami RL Altman DG van der Scheuren E for the CHART steering committee Monitoring of large randomised clinical trials: a new approach with Bayesian methods Lancet 2001 358 375 381 10.1016/S0140-6736(01)05558-1 11502318

[B12] Vahedi K Hofmeijer J Juettler E Vicaut E George B Algra A Amelink GJ Schmiedeck P Schwab S Rothwell PM Bousser M-G van der Worp B Hacke W for the DECIMAL, DESTINY, and HAMLET investigators Early decompressive surgery in malignant infarction of the middle cerebral artery: a pooled analysis of three randomised controlled trials Lancet Neurol 2007 6 215 222 10.1016/S1474-4422(07)70036-4 17303527

[B13] Fleming TR Ellenberg S DeMets DL Monitoring clinical trials: issues and controversies regarding confidentiality Stat Med 2002 21 2843 2851 10.1002/sim.1288 12325100

[B14] Cole CH Wright KW Tarnow-Mordi W Phelps DL Resolving our uncertainty about oxygen therapy Pediatrics 2003 112 1415 1419 10.1542/peds.112.6.1415 14654618

[B15] Askie LM Brocklehurst P Darlow BA Finer N Schmidt B Tarnow-Mordi W on behalf of the NeOProM Collaborative Group NeOProM: neonatal oxygenation prospective meta-analysis collaboration study protocol BMC Pediatr 2011 11 6 21235822 10.1186/1471-2431-11-6 PMC3025869

[B16] Johnston ED Boyle B Juszczak E King A Brocklehurst P Stenson BJ Oxygen targeting in preterm infants using the Masimo SET Radical pulse oximeter Arch Dis Child Fetal Neonatal Ed 2011 96 F429 F433 10.1136/adc.2010.206011 21378398 PMC3195299

[B17] Stenson B Brocklehurst P Tarnow-Mordi W for the UK and Australian and New Zealand BOOST II trials Increased 36-week survival with high oxygen saturation target in extremely preterm infants N Engl J Med 2011 364 1680 1682 10.1056/NEJMc1101319 21524227

[B18] Dixon DO Lagakos SW Should data and safety monitoring boards share confidential interim data? Control Clin Trials 2000 21 1 6 10.1016/S0197-2456(99)00042-2 10659999

[B19] Hicks LK Laupacis A Slutsky AS A primer on data safety monitoring boards: mission, methods, controversies Intensive Care Med 2007 33 1815 1818 10.1007/s00134-007-0794-9 17661013

[B20] Califf RM Harrington RA Blazing MA Premature release of data from clinical trials of ezetimibe N Engl J Med 2009 361 712 717 10.1056/NEJMsr0900910 19675335

[B21] Lilford RJ Braunholtz D Edwards S Stevens A Monitoring clinical trials - interim data should be publicly available BMJ 2001 323 441 442 10.1136/bmj.323.7310.441 11520848 PMC1121037

[B22] Chuang-Stein C Anderson K Gallo P Collins S Sample size reestimation: a review and recommendations Drug Inf J 2006 40 475 484

[B23] Borer JS Gordon DJ Geller NL When should data and safety monitoring committees share interim results in cardiovascular trials? JAMA 2008 299 1710 1712 10.1001/jama.299.14.1710 18398083

[B24] Shah SK Dawson L Dixon DO Lie RK Should sponsors and DSMBs share interim results across trials? J Acquir Immune Defic Syndr 2001 58 433 435 21937922 10.1097/QAI.0b013e318236eca3 PMC3215856

